# What Is Analysis of Covariance (ANCOVA) and How to Correctly Report Its Results in Medical Research?

**Published:** 2020-05

**Authors:** Alireza KHAMMAR, Mohammad YARAHMADI, Farzan MADADIZADEH

**Affiliations:** 1. Department of Occupational Health Engineering, School of Health, Zabol University of Medical Sciences, Zabol, Iran; 2. Razi Herbal Medicines Research Center, Lorestan University of Medical Sciences, Khoramabad, Iran; 3. Noncommunicable Diseases Research Center, Fasa University of Medical Sciences, Fasa, Iran; 4. Department of Epidemiology and Biostatistics, School of Public Health, Tehran University of Medical Sciences, Tehran, Iran

## Dear Editor-in-Chief

Sometimes in medical researches, a variable that is not among the main research variables may affect the dependent variable and its relation with the independent variable, which, if identified, can be involved in the modeling and its linear effect are controlled. This is not a dependent or independent variable, this type of variable is known as covariate (1).

To control the effect of covariate variable, not only the changes in variance of the dependent variable are examined (ANOVA), but also the relationship between the dependent variable and covariate in different levels of a qualitative variable is analyzed (Regression) (2). The statistical method that can combine ANOVA and Regression for adjusting linear effect of covariate and make a clearer picture is called the analysis of covariance (ANCOVA) (1).

ANCOVA discovers the variance changes of the dependent variable due to change in covariate variable and discriminates it from the variance changes due to changes in the levels of the qualitative variable; so it reduces the uncertain changes of the variance of dependent variable (error) and make pure results as well as increases the analytical power (3).

For example, examining the rate of learning the medical lessons in the students of different groups. The prior familiarity of some students with the topics leads to increased learning scores. Therefore, it is a covariate variable. Another example of covariate variable is a pretest score in an interventional study that needs to identify, measure and control before the intervention.

For more information on the use of the ANCOVA methodology and the appropriate way of reporting the results, note the following points:

- The dependent variable must be a continuous quantitative variable and have a normal distribution.
- The covariate must be a continuous quantitative variable (2).
- The levels of the qualitative variable must be independent (2).
- There should be a linear relationship between the dependent variable and covariate (3). If the relationship was non-linear, the Multivariate ANOVA method would be useful by considering the covariate as a secondary dependent variable another way is using linear transformations and applying ANCOVA to converted variables (3).
- The sign (+ or −) and size of the correlation coefficient between the dependent variable and covariate should be the same at each level of the qualitative variable (1). In other words, if we draw a regression line for the relationship between the dependent variable and covariate at each level of the qualitative variable, the slope of the regression lines should be the same at all levels (Homogeneity of regression slopes) (2).
- The independent variable has no relationship with the covariate variable and that it does not affect the relationship between the dependent variable and covariate (4,5).

How to correctly report ANCOVA results:

- Reporting the correlation coefficient and significant *P*-value of investigating the relationship between the dependent variable and covariate (4).
- Reporting insignificance relationship between covariate and independent variable and thus the equality of slope of regression lines.
- Provision summary table of the means of dependent variable before and after the adjustment the effect of covariate with separately reporting the p-value of means comparison.

ANCOVA is a type of ANOVA with controlling linear effect of covariate variable by using regression analysis.

Hopefully, by considering the above notes, not only researchers become more familiar with the ANCOVA method, but also the medical field studies are further enhanced by providing the appropriate results of statistical methods.
